# Traditional Chinese medicine, Qing Ying Tang, ameliorates the severity of acute lung injury induced by severe acute pancreatitis in rats via the upregulation of aquaporin-1

**DOI:** 10.3892/etm.2014.1987

**Published:** 2014-09-22

**Authors:** ZHENMING GAO, JUNFENG XU, DEGUANG SUN, RIXIN ZHANG, RUI LIANG, LIMING WANG, RONG FAN

**Affiliations:** 1 Hepatopancreatobiliary Surgery Department, The Second Affiliated Hospital of Dalian Medical University, Dalian, Liaoning 116027, P.R. China; 2 The Second VIP Ward, The Second Affiliated Hospital of Dalian Medical University, Dalian, Liaoning 116027, P.R. China

**Keywords:** aquaporin-1, qing yin tang, acute lung injury, severe acute pancreatitis

## Abstract

Aquaporin-1 (AQP-1) is expressed in lung endothelial cells and regulates water transport; thus, AQP-1 plays an important role in a number of edema-associated lung diseases. Qing Yin Tang (QYT), a traditional Chinese medicine, has been shown to effectively reduce the mortality rate of acute lung injury (ALI) induced by severe acute pancreatitis (SAP). The current study aimed to investigate the detailed mechanisms underlying the effects of QYT on ALI induced by SAP, particularly the effects on the expression levels of AQP-1 in the lung tissue. ALI was established in Wister rats who were subsequently divided into four groups: SHAM, ALI, dexamethasone (DEX) and QYT groups (n=8 per group). In the QYT group, 20 ml/kg QYT was administered by gavage immediately following the induction of SAP. Blood and lung tissues were collected 8 h following the induction of pancreatitis. The lung wet/dry ratio, as well as the levels of blood gases, serum amylase and tumor necrosis factor-α (TNF-α), were measured at 4, 8 and 12 h following SAP-associated ALI induction surgery. The expression levels of AQP-1 in the lung tissue were detected by quantitative polymerase chain reaction, immunohistochemistry and western blot analysis. No statistically significant differences were observed with regard to the levels of serum amylase, wet/dry ratio, partial pressure of oxygen, serum TNF-α and pathological changes in the pulmonary tissue between the QYT and DEX groups; however, a statistically significant difference was observed when compared with the ALI group. The expression levels of AQP-1 significantly increased (P<0.05) and lung edema was alleviated in the QYT and DEX groups, when compared with ALI group. Therefore, the expression level of AQP-1 is associated with pulmonary edema. QYT protects the lungs from injury induced by SAP via the upregulation of AQP-1, which suppresses TNF-α expression.

## Introduction

Aquaporins (AQPs) are water-selective membrane channel proteins that are expressed in numerous epithelial and endothelial cells of fluid transporting tissues, including the kidneys, eyes and lungs, where rapid regulated transport of water is required (1,2). To date, 13 AQPs have been identified in mammals, which can be subdivided into two groups based on their permeability: Seven AQPs are highly selective to the passage of water (AQP-1, AQP-2, AQP-4 and AQP-5), while five AQPs (AQP-3, AQP-7, AQP-8, AQP-9 and AQP-10) are termed aquaglyceroporins due to their ability to transport glycerol and even larger solutes (3).

AQP-1 is a 28 kD membrane-spanning polypeptide that was initially identified in red blood cells and renal tubules (4). Previous studies have revealed that AQP-1 is widely expressed in a variety of tissues, including the kidney tubules, microvascular endothelium, salivary glands and ciliary epithelium (5,6). Saadoun *et al* revealed that angiogenesis and endothelial cell migration were impaired in AQP-1-null mice, which demonstrated that AQPs play an important role in angiogenesis and the spread of tumors (7). Humans with AQP-1 mutations exhibit a urinary concentrating defect in the kidneys, indicating that AQP-1 is associated with renal function (8,9).

Approximately 20% of patients with acute pancreatitis develop severe acute pancreatitis (SAP), which has a mortality rate of ~30% (10,11). Acute lung injury (ALI) occurs as a consequence of markedly increased endothelial and epithelial permeability, with protein leakage into the alveolar space and interstitial tissues, leading to decreased gas exchange (12,13). SAP is closely associated with ALI (14); the pathogenesis of SAP-associated ALI focuses on the excessive release of cytokines and inflammatory mediators, including interleukin (IL)-1β, IL-6, IL-8 and tumor necrosis factor-α (TNF-α) (15,16). A previous study demonstrated that the expression levels of AQP-1 and AQP-5 decreased in lungs with pulmonary edema following viral infection (17).

Qin Yin Tang (QYT), a formula used in Chinese medicine, has demonstrated efficiency in reducing the mortality rate in the clinical treatment of ALI following SAP; however, the associated mechanisms remain unclear. The aim of present study was to investigate the effect of QYT on the expression of AQP-1 following the induction of SAP in the lungs.

## Materials and methods

### Animals

Male Wistar rats (weight, 200–240 g; age, 6 weeks) were purchased from the Animal Center of Dalian Medical University (Dalian, China). The Animal Research and Care Committee of Dalian Medical University (Dalian, China) approved the experimental procedures, and all animal experiments were performed under approved procedures by the Institutional Animal Use and Care Committee.

### Experimental process

QYT was provided by the Department of Traditional Chinese Medicine of the First Affiliated Hospital of Dalian Medical University. The compound was comprised of the following herbs: 15 g Herba Artemisiae Scopariae*,* 15 g *Gardenia,* 15 g *Rheum officinale* Baill*,* 9 g sodium sulfate*,* 9 g Costus root*,* 9 g Radix bupleuri*,* 9 g Rhizoma corydalis, 9 g Radix paeoniae Alba, 10 g Radix Glycyrrhizae*,* 9 g *Angelica sinensis,* 10 g Flos Lonicerae and 12 g Fructus *Forsythia.* All herbs were boiled in 300 ml water for 15 min to obtain the QYT solution.

A total of 32 rats were randomly divided into four groups, which included the SHAM, ALI, dexamethasone (DEX) and QYT groups. In clinical practise, DEX is used to alleviate the severity of pulmonary edema in pancreatitis. In the present study, it was used as a positive control to observe the therapeutic effect of QYT. The bile-pancreatic duct underwent retrograde infusion with sodium deoxycholate (15 mg/kg) to produce SAP-associated ALI in the ALI, DEX and QYT groups. The surgery was performed on the rats in the SHAM group without sodium deoxycholate. DEX (2 mg/kg) or QYT (2 ml/100 g) were administered through the femoral vein immediately following the induction of SAP in the DEX and QYT groups. Following treatment, the mice were euthanized by decapitation.

Blood and lung tissue were collected at 4, 8 and 12 h following surgery. The lung wet/dry ratio, as well as the levels of blood gases, serum amylase and TNF-α, were analyzed. In addition, the mRNA expression of AQP-1 in the lung tissue was detected by quantitative polymerase chain reaction (qPCR), while protein expression of AQP-1 was detected by immunohistochemistry and western blot analysis.

### Lung wet/dry ratio analysis

Left lung samples were excised, weighed and baked at 60°C for 24 h to obtain the dry weights. The ratio of wet to dry weight (W/D) was used as an indicator of pulmonary edema.

### Histopathological analysis

The left lower lobe was excised and inflated with 10% formaldehyde solution for 24 h. Following fixation, the lung tissue was embedded in paraffin and divided into several 5-μm sections for hematoxylin and eosin staining. A total of 10 sections were selected randomly for analysis.

Following the induction of SAP-associated ALI for 8 h, histopathology was reviewed in a blind manner using a modified histological scoring system, as previously described (8). The identifiable pathological results were scored on a scale of 0–4 as follows: 1, alveolar congestion; 2, hemorrhage; 3, leukocyte infiltration or aggregation of neutrophils in the air space or vessel wall and; 4, thickness of the alveolar wall. A score of 0 represented normal lungs; 1 represented mild ALI (<25% lung involvement); 2 represented moderate ALI (25–50% lung involvement); 3 represented severe ALI (50–75% lung involvement) and; 4 represented extremely severe ALI (>75% lung involvement) (18). An overall score of ALI was obtained based on the summation of all the scores, and the mean ± standard deviation was generated from the cohort of lung samples (three sections from each lung, eight lungs per group) at each time point to generate a cumulative histological ALI score.

### Serum amylase, arterial blood and TNF-α analysis

Amylase activity in the serum was determined by an automatic biochemistry analyzer (Hitachi 917; Boehringer Mannheim, Tokyo, Japan). Arterial blood samples were obtained from the ventral aorta of the rats. Blood gas analyses were performed using the i-STAT Portable Clinical analyzer (i-STAT Corporation, Windsor, NJ, USA). In addition, the TNF-α concentration in the supernatants was measured using a TNF-α assay kit (Nanjing Jincheng Corp., Nanjing, China), following the manufacturers’ instructions, and the measurements were expressed in nmol/mg.

### qPCR analysis of AQP-1

Tissue samples of the right lung were homogenized in TRIzol reagent (Invitrogen Life Technologies, Carlsbad, CA, USA) for total RNA isolation. cDNA was produced by reverse transcription using an RT kit (Takara Bio, Inc., Shiga, Japan), according to the manufacturer’s instructions. β-actin served as the internal control. PCR amplification of AQP-1 and β-actin was performed with SYBR Green I *Taq* Master mix (Promega Corporation, Madison, WI, USA), with cDNA synthesized from the tissues. The primers used were as follows: AQP-1 forward, 5′-ATGGCCAGCGAAATCAAGAAG-3′, and reverse, 5′-GATATCATCAGCATCCAGGTC-3′; β-actin forward, 5′-GATATCGCTGCGCTCGTCGTC-3′, and reverse, 5′-CATGAGGTAGTCTGTCAGGTC-3′. Amplification conditions were one cycle of 5 min at 94°C, 30 cycles of 40 sec at 94°C, 40 sec at the annealing temperature of 55°C and 10 sec at 72°C, followed by one cycle of 72°C for 5 min.

### Immunohistochemical analysis of AQP-1

Lung sections were stained using the streptavidin-peroxidase-biotin immunohistochemical technique. Experiments were performed following the manufacturer’s instructions (SABC kit; Boster Biological Tech Ltd., Wuhan, China). The sections were dewaxed in xylene, cultured in 3% hydrogen peroxide to eliminate intrinsic peroxidase and quenched in normal goat serum for 30 min. The sections were subsequently incubated with an anti-AQP-1 antibody (0.25 mg/ml; rabbit polyclonal; Proteintech, Chicago, IL, USA) overnight at 4°C. Phosphate-buffered saline served as the control. A biotinylated goat anti-rabbit (1:2,000; Vector Laboratories, Burlingame, CA, USA) secondary antibody was added, and the samples were incubated with an avidin-biotin complex (Vector Laboratories) for 30 min at room temperature. 3,3′-Diaminobenzidine was used for color development and hematoxylin was used for counter staining. Three representative sections from each rat were used to calculate the average staining degree for image analysis.

### Western blot analysis of AQP-1

Homogenized lung tissue was lysed on ice and the cellular plasma proteins were extracted with a protein extraction kit (Pierce Biotechnology, Inc., Rockford, IL, USA), according to the manufacturer’s instructions. Protein concentrations were determined by a Coomassie Brilliant Blue dye-binding assay. Samples (100 μg) were analyzed by SDS-PAGE and electrotransferred to a polyvinylidene diflouride (PVDF) membrane (Millipore Corporation, Billerica, MA, USA). The membrane was blocked for 1 h at room temperature with 5% skimmed milk in Tris-buffered saline with Tween-20 [TBST; 50 mM Tris-HCl (pH 7.4), 150 mM NaCl and 0.1% Tween-20]. The membrane was incubated overnight at 4°C with a rabbit anti-rat polyclonal antibody against AQP-1 (1:200 dilution) in TBST. Following washing with TBST, the PVDF membrane was incubated with a biotin-conjugated anti-rabbit antibody (GE Healthcare, Tokyo, Japan), and diluted to 1:2,000 in TBST at room temperature for 1 h. The bound antibody was detected by enhanced chemiluminescence (Amersham, GE Healthcare, Pittsburgh, PA, USA) and semi-quantitatively analyzed by densitometry with a Science Lab 99 Image Gauge System (Fujifilm, Tokyo, Japan).

### Statistical analysis

Data are expressed as the mean ± standard deviation and were analyzed by one-way analysis of variance followed by the post hoc Bonferroni test. Pearson’s correlation analysis was used to determine the association between AQP-1 expression and the degree of lung edema. P<0.05 was considered to indicate a statistically significant difference.

## Results

### Pathological changes in the lungs of all the groups

Following the induction of ALI in the rats, evident swelling and slight hemorrhage was observed in the pancreas. After 8 h, the rats in the ALI group exhibited areas of spotty or patchy necrosis in the pancreatic tissue. In the DEX and QYT groups, pancreatic necrosis and pulmonary edema were significantly alleviated compared with the rats in the ALI group. The pancreas and lungs of the rats in the SHAM group did not exhibit any marked pathomorphological changes. In the ALI group, evident inflammatory cell infiltration was observed in the interstitium of the lung under a microscope, with notable interstitial hyperemia and edema, a thickened septum and bullae formation through alveolar dilation. The inflammation was attenuated significantly in the DEX and QYT groups when compared with the ALI group. No inflammatory response was observed in SHAM group. The histopathological scores of all the groups are shown in Table I.

### Decreased W/D ratio and increased arterial blood gases in the lungs following QYT treatment

The W/D ratio, a parameter of pulmonary edema, was higher in the ALI group (P<0.01) when compared with the SHAM group (Table II). In addition, the W/D ratio decreased in the DEX and QYT groups when compared with the ALI group (P<0.05); no statistically significant difference was observed in the ratio between the QYT and DEX groups. Furthermore, marked hypoxemia existed in the ALI group, as compared with the SHAM group (P<0.01). In the DEX and QYT groups, the partial pressure of oxygen was significantly higher (P<0.05) compared with the ALI group (Table III).

### Decreased levels of serum TNF-α and amylase in the QYT group

Serum sample analysis results revealed that the serum levels of TNF-α in the ALI group were significantly higher (P<0.01) compared with those in the SHAM group at the different time points. The serum level of TNF-α in the DEX and QYT groups was significantly lower (P<0.05) compared with the ALI group. No statistically significant difference in the levels of TNF-α were observed between the DEX and QYT groups (Table IV). When compared with the SHAM group, the levels of serum amylase were significantly higher (P<0.01) in the ALI group, while the levels of serum amylase in the DEX and QYT groups were significantly lower (P<0.05) compared with the ALI group (Table V).

### Increased AQP-1 mRNA and protein expression in the QYT treatment group

**Figure 1:**
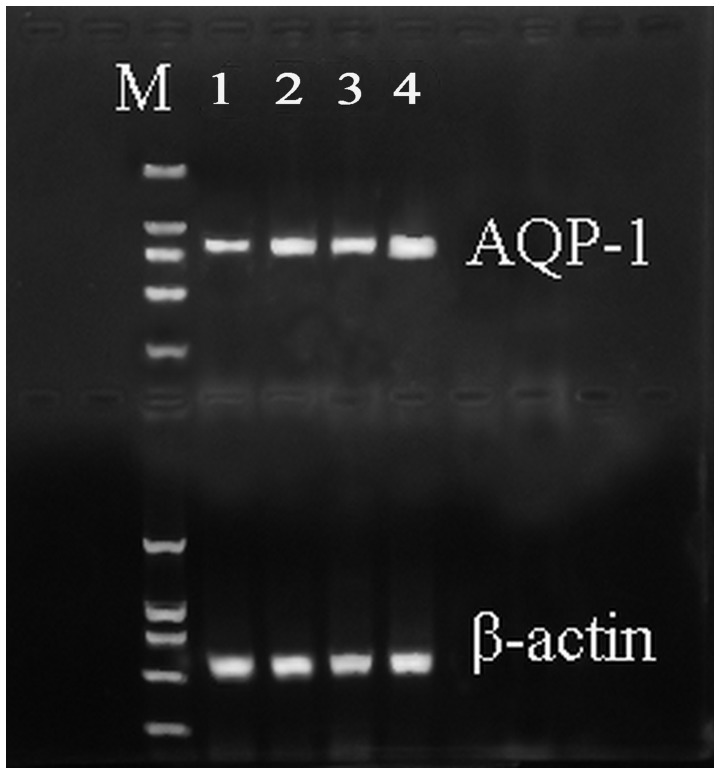
AQP-1 mRNA expression in the lungs of rats in the (1) SHAM, (2) acute lung injury, (3) dexamethasone and (4) Qing Yin Tang groups. β-actin served as the control. AQP-1, aquaporin-1.

qPCR analysis was performed to detect the expression level of AQP-1 mRNA in the four groups. The mRNA expression level of AQP-1 significantly decreased in the ALI group when compared with the SHAM group (Fig. 1). In addition, the expression levels were upregulated in the DEX and QYT groups (P<0.01) when compared with the ALI group.

**Figure 2:**
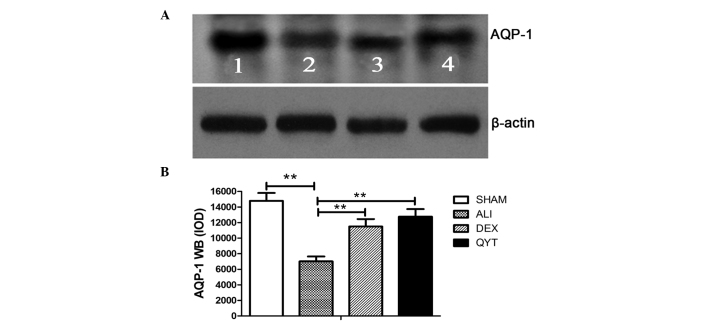
(A) Western blot analysis and (B) semi-quantitative analysis of the protein expression levels of AQP-1 in the (1) SHAM, (2) ALI, (3) DEX and (4) QYT groups. β-actin served as the control. AQP-1, aquaporin-1; ALI, acute lung injury; DEX, dexamethasone; QYT, Qing Yin Tang. ^*^P<0.05 compared to ALI group; ^**^P<0.01 compared to ALI group.

To determine whether the upregulation in the mRNA expression level of lung AQP-1 was consistent with protein expression, western blot analysis was performed to assess the protein expression levels of AQP-1 in the DEX and QYT groups. Western blot analysis demonstrated a significant increase in the protein expression levels of AQP-1 in the DEX and QYT groups when compared with the ALI group. Densitometric analysis of the 28-kD AQP-1 band revealed a significant increase in the protein expression levels of AQP-1 in the SHAM group when compared with the ALI group. The protein expression levels of AQP-1 were lower in the ALI group, but higher in the DEX and QYT groups, when compared with the SHAM group (P<0.05; Fig. 2).

### Immunohistochemical analysis of the AQP-1 protein

**Figure 3:**
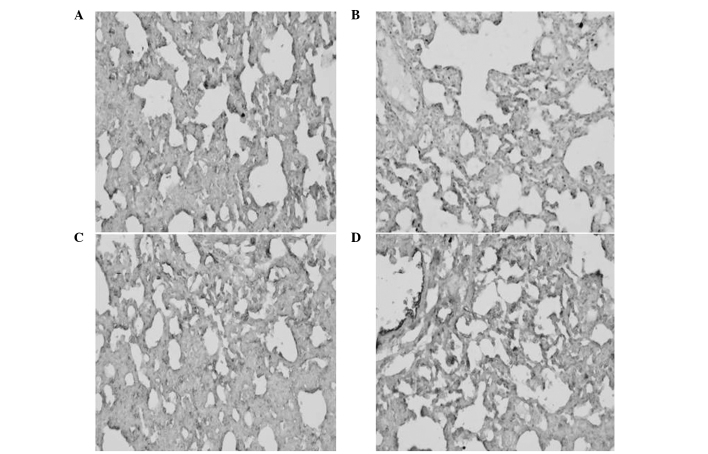
Pathological images of the lungs (magnification, ×50) in the (A) SHAM, (B) acute lung injury, (C) dexamethasone and (D) Qing Yin Tang groups.

As shown in Fig. 3, the percentage of positive AQP-1 protein expression was significantly lower in the ALI group when compared with the SHAM group (P<0.01). By contrast, the percentage of positive AQP-1 protein expression was higher in the DEX and QYT groups when compared with the ALI group (P<0.01; Fig. 3). Consistent with western blot analysis results, these observations indicate that QYT treatment effectively upregulated AQP-1 protein expression.

## Discussion

AQPs play a crucial role in maintaining water homeostasis and glycerol metabolism. Four members of the AQP protein family are expressed in the airways and lungs, and are involved in the pathological course of various types of lung disease, including pulmonary edema (19). The present study revealed that the mRNA and protein expression levels of AQP-1 were significantly downregulated (P<0.01) in rats with ALI induced by SAP, as compared with the rats in the SHAM control group. SAP induced aggravated pulmonary edema. Following treatment with DEX and QYT, AQP-1 expression was significantly upregulated (P<0.01) in the lungs with alleviative pulmonary edema, as compared with the ALI group. Previous studies have revealed that inflammatory cytokines play an important role in the pathogenesis of acute pancreatitis, with the levels of TNF-α significantly higher in patients with SAP compared with those with a mild disease at the early stage of acute attack. These studies demonstrated that TNF-α is an important index for the severity of SAP (20,21). A marked negative linear association was observed between the expression levels of AQP-1 and TNF-α in the present study. Furthermore, the results demonstrated that TNF-α regulated the expression of AQP-1 by an unknown mechanism and participated in the formation of pulmonary edema during the pathophysiological process of SAP-associated ALI development.

QYT (pancreas clearance soup) is an effective traditional prescription used for the treatment of SAP, which has advantages of a low cost and a high therapeutic effect (21). Modern clinical and experimental studies (22–24) have focused on such components, a number of which can alleviate the conditions of the patients independently. The mechanisms of alleviating SAP by QYT include improving gastrointestinal function, promoting the excretion of endotoxins and inhibiting the release of inflammatory mediums and cytokines, in order to prevent organ damage. The current study confirmed that QYT alleviated the symptoms of ALI induced by SAP. Furthermore, QYT was shown to regulate the expression levels of AQP-1. DEX, a non-specific immune inhibitor, inhibits the gene synthesis of numerous inflammatory mediators and reduces the inflammatory reaction by increasing the synthesis of anti-inflammatory proteins; thus, producing a therapeutic effect on rats with SAP (25,26). A study by Yang *et al* (27) demonstrated that DEX and QYT have a similar therapeutic effect on ALI when QYT was used to treat SAP. The incidence rates of the two severe complications, acute respiratory distress syndrome and intestinal paralysis, in the group treated with QYT were 3.6 and 5.4%, respectively, while in the control group the incidence rates were 12.7 and 18.2%, respectively (P<0.05). Furthermore, QYT effectively decreased the levels of TNF-α, IL-6 and IL-8 in patients with pancreatitis, indicating that QYT functions by downregulating the expression of TNF-α. The present study did not investigate the effect of QYT on the expression of immunoglobulin; thus, clinical studies are required for further investigation.

In conclusion, the results of the present study demonstrate that QYT upregulates the synthesis of AQP-1 by inhibiting inflammatory reactions and reducing the secretion of TNF-α; thus, alleviating ALI induced by SAP. The higher expression levels of AQP-1 in the lungs may be one of the pathogenic factors of ALI induced by SAP. Administration of QYT may reduce the extent of pulmonary edema by decreasing the expression levels of TNF-α; therefore, protecting pulmonary function.

## Figures and Tables

**Table I:** Histopathological scores in the ALI and SHAM groups.

Group	Cases, n	4 h	8 h	12 h
SHAM	8	0.33±0.58	0.34±0.46	0.41±0.58
ALI	8	1.50±0.84^a^	1.90±0.74^a^	2.08±0.64^a^
DEX	8	0.96±0.62^b^	0.87±0.48^b^	0.91±0.70^b^
QYT	8	0.98±0.43^b^	0.89±0.37^b^	0.92±0.54^b^

a P<0.01, vs. SHAM group;

b P<0.05, vs. ALI group.

ALI, acute lung injury; DEX, dexamethasone; QYT, Qing Yin Tang.

**Table II:** Lung wet/dry ratio comparison in each group following treatment.

Group	Cases, n	4 h	8 h	12 h
SHAM	8	5.76±0.45	6.38±0.52	6.09±0.28
ALI	8	10.12±0.68^a^	11.56±0.79^a^	12.49±0.61^a^
DEX	8	7.45±0.32^b^	7.03±0.56^b^	6.76±0.41^b^
QYT	8	7.46±0.29^b^	7.10±0.38^b^	6.69±0.35^b^

a P<0.01, vs. SHAM group;

b P<0.05, vs. ALI group.

ALI, acute lung injury; DEX, dexamethasone; QYT, Qing Yin Tang.

**Table III:** Arterial blood gas (mmHg) in each group of rats at different time points.

Group	Cases, n	4 h	8 h	12 h
SHAM	8	12.6±0.5	11.3±0.7	11.8±0.4
ALI	8	8.7±0.8^a^	7.9±1.1^a^	7.4±0.8^a^
DEX	8	10.6±0.46^b^	9.5±0.54^b^	9.2±0.35^b^
QYT	8	11.2±0.34^b^	9.7±0.45^b^	9.3±0.37^b^

a P<0.01, vs. SHAM group;

b P<0.05, vs. ALI group.

ALI, acute lung injury; DEX, dexamethasone; QYT, Qing Yin Tang.

**Table IV:** Serum levels of tumor necrosis factor-α in each group (nmol/mg).

Group	Cases, n	4 h	8 h	12 h
SHAM	8	71.25±5.25	74.31±6.43	78.28±5.26
ALI	8	143.47±29.00^a^	234.20±13.23^a^	273.86±14.21^a^
DEX	8	98.32±27.00^b^	110.48±32.20^b^	108.72±27.42^b^
QYT	8	98.98±23.45^b^	112.34±31.97^b^	109.67±26.44^b^

a P<0.01, vs. SHAM group;

b P<0.05, vs. ALI group.

ALI, acute lung injury; DEX, dexamethasone; QYT, Qing Yin Tang.

**Table V:** Serum amylase levels in each group (U/L).

Group	Cases, n	4 h	8 h	12 h
SHAM	8	953±88	1123±52	978±79
ALI	8	4068±361^a^	4452±348^a^	4101±432^a^
DEX	8	1231±135^b^	1312±42^b^	1128±216^b^
QYT	8	1154±141	1298±56	1084±178

a P<0.01, vs. SHAM group;

b P<0.05, vs. ALI group.

ALI, acute lung injury; DEX, dexamethasone.
